# Isolation and Genomic Analysis of the Phage vB_PaeP_fHoPae04 Infecting Pseudomonas aeruginosa

**DOI:** 10.1128/MRA.00076-21

**Published:** 2021-06-03

**Authors:** Sheetal Patpatia, Ozgenur Yilmaz, Matti Ylänne, Saija Kiljunen

**Affiliations:** a Human Microbiome Research Program, Faculty of Medicine, University of Helsinki, Helsinki, Finland; b Division of Clinical Microbiology, HUSLAB, Helsinki University Hospital, Helsinki, Finland; Loyola University Chicago

## Abstract

Here, we report the genomic sequence of Pseudomonas aeruginosa phage vB_PaeP_fHoPae04, isolated from hospital wastewater in Helsinki, Finland. The phage genome is 45,491 bp long, has a G+C content of 52.2%, and contains 70 protein-coding genes and 3 tRNA genes.

## ANNOUNCEMENT

Pseudomonas aeruginosa is a Gram-negative bacterium belonging to the ESKAPE (*Enterococcus faecium, Staphylococcus aureus, Klebsiella pneumoniae, Acinetobacter baumannii, Pseudomonas aeruginosa*, and *Enterobacter species*) group of multidrug-resistant pathogens and is one of the major causes of nosocomial infections worldwide (1–3). As P. aeruginosa is associated with various diseases and is inherently resistant to a wide range of antimicrobials (4), it has become one of the most common targets for phage therapy (5).

Phage vB_PaeP_fHoPae04 (fHoPae04) was isolated from a hospital wastewater sample collected in Helsinki, Finland, using clinical P. aeruginosa strain 6886, isolated from a nasal sample from a chronic sinusitis patient, as the host. The strain was first incubated with the wastewater sample overnight, and the phage was isolated using three rounds of plaque purification as described elsewhere (6).

Phage DNA was isolated from a freshly prepared phage lysate with a phenol-chloroform extraction and ethanol precipitation (6), and next-generation sequencing was performed at Novogene (UK). The sequencing resulted in 9,833,124 150-bp fastq reads, out of which 100,000 reads were selected for assembly. The A5-miseq integrated pipeline version 0.7.5a-r405 for *de novo* assembly of microbial genome sequences was used to assemble the phage genome (7). PhageTerm (8) was used to estimate the genome termini, and the final assembly was verified by mapping the reads back to the genome using the Geneious Prime version 2020.1.2 Assembler and Find Repeats tool (Biomatters, Ltd.). All of the 9,833,124 original reads were used for the analysis with both PhageTerm and Geneious Assembler. The genome was annotated using the Rapid Annotations using Subsystems Technology (RAST) server (9–11), tRNAscan-SE version 2.0 (12, 13), BLASTP (14), and HHpred (15). CARD (16) was used to screen the phage genome for the presence of antibiotic resistance genes, and the BLASTN program (14) was used to identify the closest genome-wide relatives of the phage. Unless otherwise stated, default parameters were used for all software tools.

The fHoPae04 genome was 45,491 bp long with a G+C content of 52.2%. The median coverage depth was 309-fold. PhageTerm did not predict clear genome termini or terminal repeats, but 183-bp direct terminal repeats were identified with Find Repeats after the reads were mapped to the assembled genome sequence. The back-mapping resulted in a circular sequence, indicating that the assembled genome was complete.

Pseudomonas phages clash (GenBank accession number MT119362) and otherone (MT119373) were the closest relatives of fHoPae04 characterized so far, both having 98% sequence coverage and 97.39% identity. This suggests that fHoPae04 belongs to the viral family *Podoviridae* and genus *Bruynoghevirus.*

The fHoPae04 genome had 70 protein-coding genes and 3 tRNA genes. Out of the 70 protein-coding genes, 49 were not assigned a function and were considered hypothetical. The 21 protein-coding genes having an identifiable function included structural and assembly proteins (such as tail and capsid proteins and terminase subunits), proteins involved in DNA replication (DNA polymerase subunits, endo- and exonucleases, and helicase), and cell lysis (lysozyme). No genes related to the lysogenic life cycle or antibiotic resistance were identified, suggesting that fHoPae04 is lytic and suitable for phage therapy.

### Data availability.

The genomic sequence of vB_PaeP_fHo-Pae04 has been deposited in GenBank under the accession number MW329986. The associated BioProject, SRA, and BioSample accession numbers are PRJNA701388, SRR13694677, and SAMN17864917, respectively.
